# Replacing Synthetic Ingredients by Sustainable Natural Alternatives: A Case Study Using Topical O/W Emulsions

**DOI:** 10.3390/molecules25214887

**Published:** 2020-10-22

**Authors:** Sara Bom, Manuel Fitas, Ana Margarida Martins, Pedro Pinto, Helena Margarida Ribeiro, Joana Marto

**Affiliations:** 1 Research Institute for Medicines (iMed.ULisboa), Universidade de Lisboa, 1649-003 Lisbon, Portugal; sarabom@campus.ul.pt (S.B.); amartins@farm-id.pt (A.M.M.); geral@phdtrials.com (P.P.); hribeiro@campus.ul.pt (H.M.R.); 2 PhD Trials, Avenida Maria Helena Vieira da Silva, n° 24 A, 1750-182 Lisbon, Portugal; mfitas@phdtrials.com

**Keywords:** synthetic, sustainable, raw materials, replacement, topical application

## Abstract

With the increasing debate on sustainability, there is a strong market trend to formulate more sustainable products for topical application. Several studies emphasize the potential applications of natural, organic, or green chemistry-derived ingredients, but comparative studies between conventional ingredients and sustainable alternatives are lacking. This type of study is considered an excellent baseline and time-saving strategy for future studies. In addition, one of the main challenges of replacing ingredients by sustainable alternatives in topical vehicles is to maintain high-quality products. Thus, the main goal of this research study was to create a well-defined strategy supported by specific experimental data for the development of sustainable topical vehicles with high-quality standards. The study was designed to evaluate the effects of replacing conventional ingredients (e.g., hydrocarbons, silicones, and preservatives) by sustainable ones on the physical, chemical, and microbiological features of topical emulsions. Additionally, in vivo assessment studies were performed to evaluate the safety, biological efficacy, and sensorial aspects of the developed formulations. The results obtained showed that the replacement of ingredients by sustainable alternatives has an effective impact on the physicochemical and structural properties of the emulsions, mainly on their rheological behavior. However, using appropriate strategies for ingredient selection and rheological adjustment, it is possible to overcome some barriers created by the use of natural raw materials, thus developing appealing and high-quality sustainable topical vehicles.

## 1. Introduction

The pharmaceutical and cosmetic industries have been facing increasing pressure to design and launch sustainable products. In the last few years, these industries have been trying to rapidly reinvent themselves, to produce natural, organic, and eco-friendly formulations. Lately, there is also a notorious concern regarding the responsible and ethical supply of raw materials, the practice of fair trade, control of emissions and waste, use of recycled and/or recyclable packaging, as well as the increase of the biodegradable content of the formulas. In the short term, the main goal of any corporation that is trying to implement the concept of sustainability across the product life cycle is the reduction of the environmental, social, and economic impacts while strengthening its position in the market [1,2].

Although advances in the direction of sustainable production are already visible, the selection of raw materials that fulfill all sustainability requirements continues to be a challenge for formulation development processes. A raw material can only be considered sustainable if it has environmentally preferable attributes that meet ethical, social, and economic responsibilities, as previously described [1]. Accordingly, there are five key points that should be considered when selecting sustainable raw materials: (i) the biodegradability pattern and the bio-based composition of the ingredients; (ii) the origin and the source of the raw material, which is as important as the way it was synthesized, extracted, and/or purified; (iii) a natural, green, and/or organic ingredient cannot automatically be classified as sustainable; (iv) a synthetic raw material can be considered sustainable when compared to other options in the market; and (v) sustainability does not only pertain to environmental impact but, also, to economic and social domains [2]. Considering the last key point, it is also necessary to consider the quality of the final product, as this will have a considerable impact on the economic and social domains.

Several studies have described different strategies towards sustainability, such as the exploitation of local and low-cost natural ingredients (e.g., pine nut derivatives and rice water), the production of new and/or improved raw materials through green chemistry, and the use of industry redundancies and/or wastes (e.g., spent coffee grounds and cork) [1,3,4,5,6,7,8,9,10]. Nevertheless, the inclusion of this type of ingredient requires further investigation concerning the physicochemical impact of its use [1]. From this point of view, the buzzwords related to sustainable topical products need to be expanded beyond safety and include efficacy, quality, and marketability. One of the main challenges of replacing synthetic ingredients with sustainable alternatives in topical vehicles is to maintain the high-quality standards normally associated with unsustainable products, as the consumer expects similar quality and effectiveness [11]. From a technical point of view, replacing high-performing synthetic raw materials with sustainable natural ingredients can be quite challenging due to instability and aesthetic limitations normally associated with their use [11,12]. Thus, understanding the chemical and physicochemical properties of these compounds plays a crucial role in the formulation process, since it allows predicting stability, performance, and aesthetic matters [1,13]. Alongside, formulators are continuously searching for instrumental methodologies and tools to evaluate the sustainable performance of raw materials and final products [2]. However, there is a lack of comparative studies between conventional ingredients and sustainable alternatives. These studies can also be considered excellent baselines and time-saving strategies for future works.

Natural oils, butters, fats, or waxes, for example, are ingredients to be considered for the replacement of synthetic emollients [1]. The use of this type of ingredient may have several disadvantages, such as the occurrence of crystallization when using natural triglycerides, oxidation with unsaturated compounds, the development of undesirable colors or odors, and incompatibilities between natural and synthetic ingredients that can affect the stability of the formulations [13]. For the replacement of synthetic preservatives, natural and nature-identical substances can be used, as well as self-preservation techniques or “booster” ingredients. Among the several preservation systems available to substitute the conventional ones, the choice of the most sustainable option is not consensual due to environmental, health, and/or formulation limitation issues associated with this type of ingredient [2].

The study reported herein aimed to replace synthetic ingredients used as emollients (e.g., hydrocarbons and silicones) and preservatives (e.g., phenoxyethanol) by sustainable ingredients and to evaluate the influence of the replacement on the physical, chemical, and microbiological features of the emulsions they are part of. The safety, biological efficacy, and sensorial aspects of the developed topical formulations were also assessed by in vivo studies. Additionally, an adequate scale-up procedure was designed; the critical points of the manufacturing process were identified; and the physical, chemical, and microbiological stabilities of the final formulations were also analyzed.

## 2. Results and Discussion

### 2.1. Raw Materials Selection

Considering the life cycle of a cosmetic product, the design and selection of raw materials are considered the phases with the highest impacts on sustainability, as previously described by our group [2]. Furthermore, and considering the high-quality standards, selecting raw materials with physicochemical properties similar to those of conventional synthetic ingredients is preferable for an effective replacement process [1]. Table 1 presents a description of the selected alternative ingredients to be used, including their categories. Two major selection criteria were used to select the petrolatum and dimethicone alternatives: sustainability and identical physicochemical properties. For petrolatum (P) selection, 11 alternatives were considered, including one petrolatum like-ingredient, four semisolid butters, two jelly-like ingredients, one wax-like ingredient, and three blends of ingredients. Among the selected options, LB (*Prunus amygdalus dulcis* oil, hydrogenated vegetable oil, and *Citrus limon* peel oil); KV (*Ricinius communis* seed oil, hydrogenated *Rhus verniciflua* peel wax, *Rhus succedanea* fruit wax, ascorbyl palmitate, and tocopherol); OB (hydrogenated olive oil and *Olea europaea* fruit oil); MB (*Magnifera indica* seed butter); SB (*Butyrospermum parkii*); and NVA (*Ricinus communis* seed oil, hydrogenated castor oil, and *Copernicia cerifera* cera) seem to be the best alternatives for the replacement of petrolatum, in terms of rheological behavior. NVA, an emollient of vegetable origin, although having a chemical structure fundamentally different from petrolatum, was designed and launched in the market as a direct alternative, since it reproduces the texture, the appearance, and the after-feel physicochemical properties of the original synthetic raw material. SB is considered one of the most sustainable natural resources in the world; it is associated with certified fair trade and is still recognized for its excellent qualities as a skin and hair conditioner and its antiaging, UV-protective, and anti-inflammatory properties [14,15,16]. LB, MB, and OB are of organic origin and have extraordinary moisturization properties, already duly proven. KV, which is a blend of natural waxes and oils, shows excellent skin-feel properties, and, although chemically different, its macroscopic appearance is similar to that of petrolatum. On the contrary, AP (PEG-8 beeswax) was considered the “worst” alternative for the substitution of petrolatum due to its physicochemical features. This raw material was selected, and its behavior was studied to understand and define what types of rheological manipulation strategies are necessary to minimize the replacement impact. Additionally, four other options were considered, namely OJ (ascorbyl palmitate, cera alba, *Copernicia cerifera* cera, *Ricinus communis* seed oil, and tocopherol); LPC (C10-18 triglycerides); AC (jojoba esters, *Helianthus annuus* seed wax, *Acacia decurrens* flower wax, and polyglycerin-3); and CM (glyceryl dibehenate, tribehenin, and glyceryl behenate). These alternatives were selected to understand the influence of incorporating blends of ingredients in the rheological and sensory features of the final product. Overall, the selection strategy was based on a previous work developed by our group [1,2] and on the works of Garrison and Dayan [13] and Beerling [17] which, in their reported studies, chose different types of oils, butters, fats, and waxes and interlinked the chemistry and the physicochemical properties of the raw materials with the advantages and limitations of their use. 

Concerning dimethicone (D) alternatives, five ingredients were tested, namely PLS (hydrogenated ethylhexyl olivate and hydrogenated olive oil unsaponifiables), EMG (C15–19 alkane), MD (octyldodecyl myristate), DPPG (propylene glycol dipelargonate), and SQ (hydrogenated polysobutene). The choice of these ingredients was based on their sustainability and their different viscosities (high, medium, and low), which were considered. D and its alternatives are in completely different viscosity ranges. D is classified as a very high-viscosity raw material, while the alternatives can be classified according to the scale presented in the Evonik’s product catalog: EMG and DPPG—low viscosity (5–10 mPa.s), PLS—medium viscosity (10–20 mPa.s), and MD and SQ—high viscosity (20–50 mPa.s).

For the selection of preservatives to replace phenoxyethanol (PHE), parameters related to the environmental impact, efficacy, and low potential for sensitization were considered. GE (benzyl alcohol, salicylic acid, glycerin, and sorbic acid) was equated, because it has a wide range of global regulatory acceptance; is COSMetics Organic Standard (COSMOS)-accepted and meets the ECOCERT standards; has a broad spectrum activity on bacteria, yeast, and molds; is water-soluble; and has a wide pH compatibility (pH 3–8) and low odor and color. SC2 (sodium benzoate and potassium sorbate) and SC3 (dehydroacetic acid and benzyl alcohol) were considered as possible alternatives, because they are broad-spectrum preservatives with improved ecotoxicity and low potential for sensitization. GU (gluconolactone and sodium benzoate) was chosen, because it has a wide range of global regulatory acceptance; is ECOCERT/COSMOS-accepted, NATRUE-approved, and Soil Association-approved; and has broad applicability and spectrum activity, as well as moisturization benefits. SA (sorbic acid) and SOB (sodium benzoate), which are food-approved preservatives, were also considered, because benzoic acid, sorbic acid, and their salts are compliant and allowed in topical products certified as natural and organic, according to the ECOCERT and COSMOS standards.

### 2.2. Formulation’s Design

For the development of a sustainable topical vehicle, a standard oil-in-water (O/W) emulsion containing 10% of petrolatum (P), 5% of dimethicone (D), and 1% of phenoxyethanol (PHE) was used as a control and as a starting point for the development of the subsequent ones. All possible combinations between the 11 alternatives to replace P, the 5 alternatives selected for D replacement, and the 6 alternatives to replace PHE were tested. Then, a decision-making scheme was used to validate and/or exclude the formulations developed in terms of the physicochemical properties (see Section 3.2.4), and, based on results obtained in the pre-formulation studies (data not shown), two formulations (F1 and F2) were characterized and selected for further assessment. Concerning the composition, the most significant difference between these two O/W emulsions was the incorporation of NVA (F1) and AP (F2) as petrolatum alternatives, while PLS and GU were selected as those that had the least physical impact to replace dimethicone and phenoxyethanol, respectively. In both formulations, it was necessary to increase the percentage of surfactant to solve the stability issues and use different strategies for adjusting the viscosity. To increase the viscosity of the formulations and strengthen their structure, a specific percentage of stearyl alcohol was added depending on the necessity of each formulation, as well as 0.5% of Siligel™ (SG—xanthan gum, lecithin, sclerotium gum, and pullulan), a natural gelling agent. The increase in viscosity was different for the two formulations, given the chemical and structural differences between AP and NVA, already mentioned above. Tegosoft^®^ MM (myristyl myristate) was also added to the formulation with NVA (F1) to a final recommended concentration of 2%. This natural and renewable-based wax produced by an enzymatic process is recognized for its ability to add body to O/W emulsions while leaving a pleasant soft skin feel. Regarding the strategies employed for solving stability issues and improving the performance of the formulations, many others could have been chosen, but the aim was not to drastically change the quantitative and qualitative composition of the control formulation. In fact, one of the goals of this research work was to prove that it is feasible to perform high-quality reformulation processes without changing drastically the composition of the original formulas and, at the same time, guarantee consumer acceptance.

### 2.3. Formulation’s Physicochemical Characterization

The physicochemical characteristics of the validated formulations (F1 and F2) were then determined. Concerning the macroscopic organoleptic characteristics, the formulations showed a homogeneous appearance with bright white color, similar to the control formulation, and none of them showed phase separation in the centrifugation tests. The pH values were adjusted for both formulations (pH range 5.50–7.15), and no significant variations were observed over time, supporting the use of the sodium citrate buffer as an effective strategy. 

**Figure 1:**
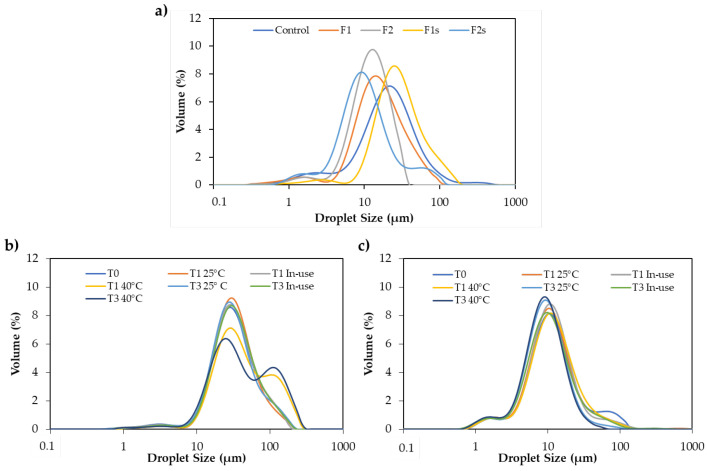
(**a**) Droplet size distribution of lab scale (F1 and F2) and pilot lab scale (F1s and F2s) formulations after manufacturing; *n* = 6; (**b**) Droplet size distribution of formulation F1s at the three different evaluation time points and storage conditions; *n* = 6; (**c**) Droplet size distribution of formulation F2s at the three different evaluation time points and storage conditions; *n* = 6.

The droplet size of the emulsions was used as an additional indicator of physical stability. The stability is influenced by the droplet size and distribution profile of oil droplets, with higher stability achieved in emulsions with smaller droplet sizes and tighter size distributions [18]. The droplet size distribution also directly influences aspects such as the degradation rate, long-term stability, resistance to creaming, texture and optical appearance, viscosity, physiological efficiency, and chemical reactivity [19]. The stability of the emulsions is also dependent on the density difference between the droplets and the medium, and instability can be manifested by physicochemical processes such as creaming or sedimentation, flocculation, Ostwald ripening, coalescence, and phase inversion [18,20]. In terms of droplet size distribution, F1 and F2 presented a monomodal population with a distribution profile comparable to the control (Figure 1a). However, by adopting a critical analysis approach, it is possible to conclude that the formulation F2 is the most stable, since the droplet size is smaller, and the distribution profile is tighter when compared to the control and formulation F1. The results obtained for formulation F2 suggest that wax-like ingredients with self-emulsifying capacity are preferable to substitute petrolatum in terms of physical stability. These results also suggest that effective raw material substitutions do not have a critical influence on the droplet size distribution.

**Figure 2:**
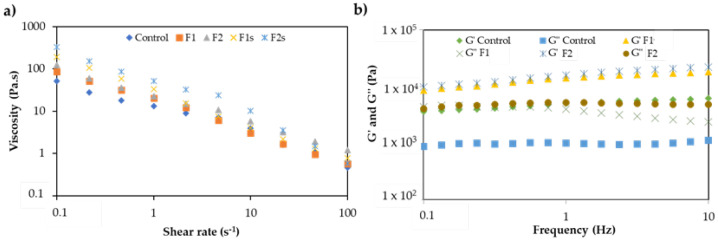
(**a**) Viscosity flow behavior of lab scale (F1 and F2) and pilot lab scale (F1s and F2s) formulations after manufacturing, and (**b**) viscoelastic behavior of lab-scale emulsions (F1 and F2).

As shown in Figure 2a, F1 and F2 have similar rheological profiles compared to the control. The differences obtained for the first five viscosity values may result from the addition of the gelling agent. These results show that the structure of the formulations F1 and F2 are more resistant to breakdown than the control. The viscosity results obtained for F1 are in accordance with what was expected since P was replaced by NVA, which can be considered a direct replacement alternative, since the physicochemical behavior is equivalent. Formulation F2 contains AP instead of P, and the AP chemical structure influences the behavior to the torque response, given that AP is a wax-like ingredient and more resistant to structural breakdown. Despite the differences in viscosity, all the formulations exhibited a similar behavior to the torque response (i.e., similar flow curve), with the apparent viscosity decreasing concomitantly with the increase in shear rate. Thus, the results emphasize the success of the strategies adopted to minimize the impact of the hydrocarbons and preservative replacement. To infer about the viscoelastic behavior of the emulsions, oscillatory tests were performed, and the results showed that the elastic module was higher than the viscous module (G′ > G″) for all formulations, meaning the formulations present a strong network (“solid-like”) with higher stability (Figure 2b). These results agree with previously described ones [21], since these formulations are considered as structured semisolid emulsions. Formulations F1 and F2 present higher elastic and viscous modules than the control, meaning these formulations are slightly more structured, which can influence the spreading behavior on the skin.

The evaluation of the adhesive strength was performed at two different temperatures, 25 °C and 32 °C (Table 2). The lower temperature was used to assess how the formulations behave in terms of adhesion when removed from the packaging, while the higher temperature was used to perceive the behavior of the same when applied to the skin. At 25 °C, F1 showed adherence and tackiness properties similar to the control, while F2 showed slightly higher values. At 32 °C, both formulations seemed to behave like the control. When comparing the results, it was also noticeable that the adhesion and tackiness decreased with the increasing temperature. Furthermore, the F2 formulation showed a more significant oscillation of values between the two analysis temperatures, and at 25 °C, it was less comparable to the control, suggesting that the incorporation of a temperature-sensitive raw material—in this case, AP—may have an impact on this type of sensory property.

The spreadability of cosmetic emollients can be defined as their capability to cover an area of the skin over time and is considered a determining factor for the consumer’s acceptance of cosmetic emulsions [14,22]. According to published research, some physicochemical properties of emollients (i.e., viscosity, polarity, and physical state) may affect the mechanisms of interaction of the product with skin, the structural organization, and the organoleptic characteristics of the emulsion. Specifically, the physical state of the emollients can influence the viscoelastic behavior and, consequently, the emulsions’ spreadability on the skin [23,24]. In terms of spreading, formulation F2 exhibited a behavior similar to the control, while F1 showed a higher spreadability than the control when using a glass surface. As mentioned previously, formulations F1 and F2 showed differences in terms of viscoelastic behavior when compared to the control, which can be linked to the physical state of the emollient alternatives incorporated in the emulsions. Furthermore, the viscosity of the emulsions, ranging between 100–1000 s^−1^, could be associated to the spreading or viscosity of the product during application [23]. The viscosity data obtained within this range confirmed the spreading data. 

Overall, the results obtained support the selection of formulations F1 and F2 to proceed to subsequent steps, since they mirror an effective and successful replacement of petrolatum, dimethicone, and phenoxyethanol. 

#### Scaling Up and Stability

Emulsions are thermodynamically unstable, which is why increasing the batch scale is a challenging step within the development process. Increasing the scale of the emulsions is extremely important, since many process limitations, which are not detectable on a small scale, can become significant when transposing to a larger scale. In addition, when increasing the batch size, changes in the behavior and efficiency of the ingredients to be mixed may occur. The transition from the laboratory production scale to industrial production is not straightforward, and the product is normally manufactured at intermediate scales, larger than the initial ones but smaller than the industrial scale. This procedure aims to simulate high-volume production in order to optimize operational parameters and guarantee product quality. 

In this research work, the scale-up production was carried out by increasing the volume of the lab scale by ten-fold (from 100 g to 1 kg). The pilot lab-scale emulsions (F1s and F2s) were analyzed for pH, macroscopic organoleptic characteristics, physical stability, droplet size, and rheological properties. Concerning the macroscopic organoleptic characteristics, formulation F1s and F2s presented a homogeneous appearance identical to the lab-scale ones, with no visual sign of instability. These results were confirmed by centrifugal tests where no phase separation was observed for any formulation. In the case of the lab-scale formulation, the different process parameters (stirring rate) may impact the physicochemical characteristics, such as droplet size. The results obtained for droplet size distribution showed a monomodal population for formulation F1s at time zero (after manufacturing), which is in accordance with the results obtained for the lab scale formulation (Figure 1a,b). However, the mean droplet sizes (90% of the droplets; d90) for the two lab scales at time 0 were significantly different (43.75 ± 0.64 μm and 76.84 ± 0.70 μm for lab scale and pilot lab scale, respectively). The formulation F2 presented a monomodal population with a d90 of 24.98 ± 0.06 µm, whereas, after the pilot lab-scale production, the formulation F2s presented a bimodal population with a d90 of 34.31 ± 1.32 µm (Figure 1a,c). Thus, the scale-up process seems to influence the droplet size distribution of the formulations, with the droplet size significantly increasing when the production scale is increased. The viscosity of formulation F1s (12.23 to 15.56 Pa.s) and F2s (14.97 to 32.32 Pa.s) increased significantly at time zero, compared to the results obtained for the lab-scale emulsions. The obtained viscosity results clearly showed that the scale-up had a positive influence on the quality and performance of formulation F1.

Introducing a topical product on the market involves several phases of research, including the analysis of different aspects of the stability of the emulsions. The purpose of stability testing is to ensure that a cosmetic product meets the intended physical, chemical, and microbiological quality standards, as well as functionality and aesthetics, when stored under appropriate conditions. To study these effects, stability tests were performed at different time points under three different storage conditions (Table 3). 

Regarding pH, the values remained constant over time for both formulations. Concerning the viscosity, the results showed slight oscillations along the time, independently of the storage conditions. These oscillations are probably due to structural rearrangements. Both formulations contained polymers (xanthan gum, lecithin, sclerotium gum, and pullulan) that are normally sensitive to dry and/or humid conditions, which could affect the viscosity of the final products [25]. Furthermore, the apparent viscosity values provided a comparison of the resistance to structural breakdown between the emulsions. As the production scale increased, the resistance to structural breakdown slightly increased. The droplet size for F1s stored at 25 °C and at 25 °C (in-use) did not vary over time. However, when stored at 40 °C, there was an alteration in the droplet size distribution, indicating possible physical instability (Figure 1b). The droplet size for F2s stored at the three different conditions did not vary over time, which is a suitable sign of physical stability (Figure 1c). The usual batch-to-batch variations were not analyzed. Nevertheless, the overall results suggest that the emulsions produced in a pilot lab-scale had a similar physical stability compared to the lab-scale ones. 

Microbiological testing revealed that the two formulations were microbiologically stable over time independently of the storage conditions, since the total aerobic mesophilic bacteria, yeast, and mold count values were considered acceptable, according to the established criteria (<10^2^ colony-forming units (CFU)/g) for these products (Category 2). The results also showed total absence of *Pseudomonas aeruginosa*, *Staphylococcus aureus*, *Candida albicans*, and *Escherichia coli*. Furthermore, the results obtained are within the specifications for criterium A; thus, the microbiological risk is considered tolerable, and the cosmetic product is deemed to meet the requirements of this international standard without additional rationale. A preliminary study was also conducted to evaluate if the packaging material had an influence on the microbial growth, a necessary step in the selection process of packaging for sustainable topical products. The results showed that the packaging materials under study had no influence on the microbial growth, so only sustainability and use issues were considered for the final choice of packaging. Certified plastic and aluminum packages were chosen due to the sustainability features previously highlighted [1]. Glass could also have been a possible choice but was not considered due to the transportation logistics and increased weight of each order. Considering the advantages and disadvantages of using plastic or aluminum packaging, the final choice was to use low-density polyethylene (LDPE) tubes, because, functionally and aesthetically, they were more suitable for the products in question.

### 2.4. In Vivo Studies 

In the Human Repeat Insult Patch Test (HRIPT), no reactions were observed in the initial three weeks of contact or after the final challenge contact. Therefore, the repeated application of the products on the skin of the volunteers did not induce any sensitization, and the formulations showed good skin compatibility. Thus, formulations F1 and F2 have low potential for irritation, sensitization, and allergic contact.

**Figure 3:**
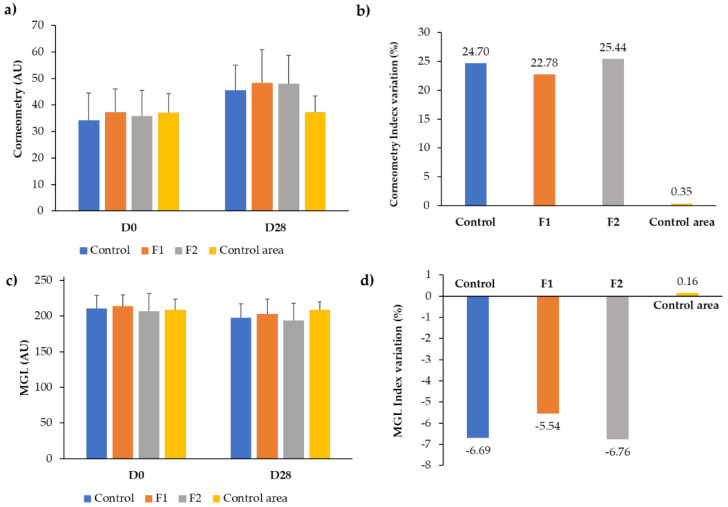
Skin hydration results using the Corneometer and MoistureMap MM 100 devices. (**a**) Corneometry results over time (*n* = 9 ± SD). (**b**) Corneometry index variation vs. D0 (%). (**c**) Mean grey level (MGL) results over time (*n* = 9 ± SD). (**d**) MGL index variation vs. D0 (%).

Two complementary 2D skin hydration methods were used to assess the hydration performance of the moisturizer formulations developed. The results obtained were graphically represented as absolute values ± SD and as index variation vs. D0 (%)—Figure 3. An excellent correlation between the use of the Corneometer device and the mean grey level (MGL) parameter extracted from the MoistureMap MM 100 device was obtained. The results further established that the daily application of the three formulations increased the moisture content (i.e., higher corneometry values and lower MGL values) and that the formulations F1 and F2 had similar behaviors to the control. The results obtained for the control area showed no significant differences in hydration between days 0 and 28, as expected. 

**Figure 4:**
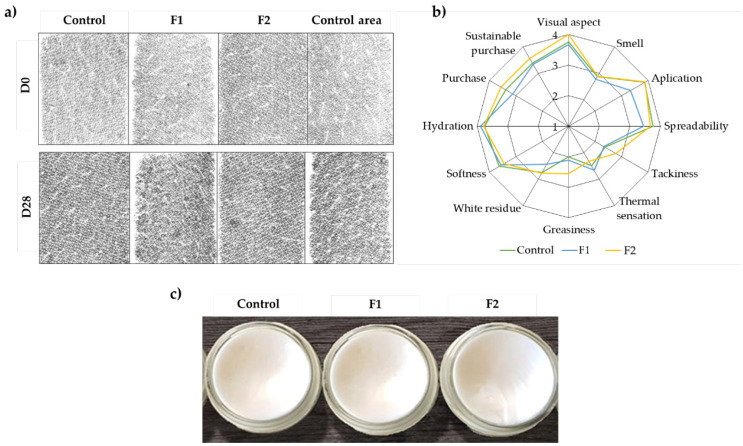
(**a**) Hydration images of the skin on the volar side of the forearm of one of the volunteers extracted from the MoistureMap MM 100 device before and after application. (**b**) Sensory profiles. In vivo assays (*n* = 9). (**c**) Macroscopic aspect of the validated formulations and control.

The skin hydration imaging method quantifies skin surface hydration, also allowing visualization and further analysis of the skin micro-relief or topography. The images of the sensor give graphical information on the near surface hydration distribution and the micro-topography of the skin tissue. The capacitance values are coded in a range of 255 gray levels (0 = black and 255 = white), thus the skin hydration can be characterized using parameters derived from gray level histogram using specific software [26,27]. Figure 4a shows an example of the capacitance images of the skin obtained during the test, where it was possible to compare the skin hydration level before (D0) and after application (D28). The grayscale values decreased significantly after 28 days of application, and the topography of the skin presented less irregularity, suggesting a good hydration capacity for the products being tested. These results are in accordance with the quantitative data.

The sensorial analysis plays an essential role in understanding the acceptance of the product by the consumer; therefore, it is essential to evaluate the product´s sensory attributes before and during application (Figure 4b). For this test, the volunteers who participated in the efficacy evaluation were selected, given that they applied the three formulations (Control, F1, and F2) for 28 days, so their evaluations were considered more reliable. Regarding the visual aspect (Figure 4c), all the volunteers agreed that formulation F2 was the best, assigning the maximum score. Formulation F1 and the control were scored as also having good visual appearances, however, with a slight difference from F2. Concerning odor, the volunteers classified all formulations as equally pleasant, meaning that the replacement of the ingredients was successful and did not affect the smell. Differences in smell are expected when replacing synthetic ingredients with natural and sustainable raw materials, which normally have more pronounced odors. However, odor was one of the selection criteria for the final formulations, which excluded the ones with unacceptable odors. In terms of application and spreading, the volunteers considered that formulation F2 was easy to apply and fast to spread, similar to the control. Formulation F1 was considered slightly more difficult to apply and spread. This formulation was considered the most similar to the control in terms of viscosity and spreading (see Section 2.3), but, in terms of sensorial analysis, the volunteers considered it more fluid, even if it took a longer time to be absorbed by the skin. Concerning tackiness and greasiness, F1 showed a similar score to the control, with the volunteers considering that these formulations did not leave a tacky and/or oily feeling after application. Regarding thermal sensation (1—cold and 4—hot), all the volunteers felt a cold sensation upon application of all the formulations. None of the formulations under testing left a white residue during and after application, and the results were coherent with the ones obtained for application and spreadability. Regarding softness and hydration, the volunteers unanimously considered that the three formulations hydrated the skin, leaving it soft. To conclude, the volunteers answered a final question about the probability of acquiring the products in question. The answers clearly showed that the volunteers preferred formulation F2 to the control and, finally, F1. To evaluate if the volunteers would equate a more ethical consumerism when buying topical products, they were also asked: “Would you buy the product knowing that it contributes to sustainability, even if in terms of performance, you did not like 100%?” The answers showed that, although the volunteers were concerned with sustainability, the performances of the products continued to be a decisive factor at the time of choice and purchase. 

## 3. Materials and Methods

### 3.1. Materials

For the preparation of emulsions, the following excipients were used: solid Vaseline (petrolatum) was obtained from LABCHEM (Zelienople, Pennsylvania, USA); Apifil^®^ (PEG-8 Beeswax) was obtained from Gattefossé (Nanterre, France); and natural Vaseline Type A (*Ricinus communis* seed oil, hydrogenated castor oil, and *Copernicia cerifera* cera) and Natura-Tec plantsil (hydrogenated ethylhexyl olivate and hydrogenated olive oil unsaponifiables) were obtained from Natura-Tec^®^ French Riviera (Fréjus, France). ABIL^®^ 350 (dimethicone) was obtained from Evonik Industries AG Personal Care (Essen, Germany). Versatil^®^ PC (phenoxyethanol and caprylyl glycol) was obtained from Evonik Dr. Straetmans GmbH (Hamburg, Germany). Geogard Ultra™ (gluconolactone and sodium benzoate) was obtained from Lonza (Basel, Switzerland). TEGO^®^ Alkanol 18 (stearyl alcohol) was obtained from Evonik Industries AG Personal Care (Essen, Germany). d-Panthenol (panthenol) was obtained from DSM Nutritional Products Europe Ltd. (Basel, Switzerland). Tegosoft^®^ MM (myristyl myristate) was obtained from Evonik Nutrition & Care GmbH (Essen, Germany). Siligel™ (xanthan gum, lecithin, sclerotium gum, and pullulan) was obtained from Lucas Meyer Cosmetics (Champlan, France). Essencia Delicate 351240 was obtained from Iberchem (Murcia, Spain). Oxynex^®^ 2004 (propylene glycol, butyl hydroxytoluene (BHT), ascorbyl palmitate, glyceryl stearate, and Citric acid); citric acid; sodium citrate dehydrate; and sodium hydroxide were obtained from Merck KGaA (Darmstadt, Germany). Lactic acid was obtained from AppliChem (Barcelona, Spain). Purified water was obtained by reverse osmosis and electrodeionization (Millipore, Elix 3) and was filtered (pore 0.22 µm) before use.

### 3.2. Methods

#### 3.2.1. Selection of Petrolatum, Dimethicone, and Phenoxyethanol Alternatives

For the selection of emollients, two major criteria were used to decide on petrolatum and dimethicone alternatives: first, the sustainability of the choices, and second, the choice of raw materials with physicochemical properties identical to those of synthetic origin. The selected petrolatum alternatives included vegetable and/or animal waxes, oils, and mixtures of waxes and oils, while alternatives to dimethicone only included ingredients of vegetable origin. Eleven alternatives for petrolatum were selected: LB—*Prunus amygdalus dulcis* oil, hydrogenated vegetable oil, and *Citrus limon* peel oil; KV—*Ricinus communis* seed oil, hydrogenated *Rhus verniciflua* peel wax, *Rhus succedanea* fruit wax, ascorbyl palmitate, and tocopherol; OB—hydrogenated olive oil and *Olea europaea* fruit oil; MB—*Mangifera indica* seed butter; NVA—*Ricinus communis* seed oil, hydrogenated castor oil, and *Copernicia cerifera* cera; SB—*Butyrospermum parkii*; AP—propylene glycol dipelargonate; OJ—ascorbyl palmitate, cera alba, *Copernicia cerifera* cera, *Ricinus communis* seed oil, and tocopherol; LPC—C10-18 triglycerides; AC—jojoba esters, *Helianthus annuus* seed wax, *Acacia decurrens* flower wax, and polyglycerin-3; and CM—glyceryl dibehenate, tribehenin, and glyceryl behenate.

Five alternatives for dimethicone were selected: PLS—hydrogenated ethylhexyl olivate and hydrogenated olive oil unsaponifiables, EMG—C15-19 alkane, MOD—octyldodecyl myristate, DPPG—propylene glycol dipelargonate, and SQ—hydrogenated polyisobutene.

For the preservative selection, parameters related to environmental impact, efficacy, and low potential for sensitization were considered. Six alternatives for phenoxyethanol were selected: GE—benzyl alcohol, salicylic acid, glycerin, and sorbic acid; GU—gluconolactone and sodium benzoate; SC2—sodium benzoate and potassium sorbate; SC3—dehydroacetic acid and benzyl alcohol; SA—sorbic acid; and SE—sodium benzoate.

#### 3.2.2. Design and Manufacturing Process of Lab-Scale Formulations

O/W emulsions were prepared by mixing the oil and aqueous phases in different stainless-steel bowls and melting at 75 °C in a water bath (digital water bath Model 601, Nahita). The emulsification phase was performed by slowly adding the oil phase to the aqueous phase with high shear mixing at a rate about 11,000 rpm/min (IKA T25 ULTRA TURRAX) for 1 min, followed by manual continuous stirring until room temperature. A sodium citrate buffer at a concentration of 1.5× the amount of Geogard Ultra^®^ (gluconolactone and sodium benzoate) was added to the oil phase to maximize the pH stability of the final formulations. Additionally, the perfume and provitamin B5 (d-panthenol) were added at 30 °C. All formulations were prepared in batches of 100 g.

#### 3.2.3. Physical and Chemical Characterization of Emulsions

The macroscopic appearance of each formulation was visually analyzed and used as a first stability indicator. The pH was determined and controlled using a digital pH meter with a glass electrode (SevenEasy™ by Mettler Toledo (Columbus, OH, USA). The third stability test—accelerated stability test—evaluated the behavior of the samples (2 g) under centrifugation (Medifuge™ Small Benchtop Centrifuge by Haraeus (Hanau, Germany)) during three periods of 5 min at 4000 rpm and 25 °C. Centrifugal tests were performed one day after preparation of the emulsions (*t* = 24 h).

The droplet size distribution of the emulsions was measured by light scattering using a Malvern Mastersizer 2000 (Malvern Instruments, Worcestershire, UK) coupled with a Hydro S accessory. For a correct turbidity, about 0.5 g of each validated formulation, corresponding to an obscuration between 10% and 20%, was added to the sample chamber containing 120–150 mL of water and stirred at 1750 rpm. The data was expressed in terms of relative distribution of the volume of droplets and given as diameter values corresponding to percentiles of 10%, 50%, and 90% (mean ± SD, *n* = 6). Measurements were performed one day after preparation of the emulsions (*t* = 24 h).

Structural experiments were performed with a controlled stress Kinexus Rheometer (Malvern Instruments, Worcestershire, UK). Rotational viscosity was determined using cone-and-plate geometry (truncated angle 4° and radius 40 nm). Dynamic viscosity measurements were carried out between 1 and 1000 Pa on a logarithmic increment, ranging from 0.1 to 100 s^−1^. The measurements were performed at 25 °C 3 days after preparation of the emulsions (*t* = 72 h). Oscillation frequency sweep tests were performed at frequencies ranging between 0.01 and 1 Hz using cone-and-plate geometry (truncated angle 4° and radius 40 nm). All measurements were performed at 25 °C at *t* = 72 h. To evaluate tackiness and adhesion properties, a pull-away test was performed at two different temperatures, 25 °C and 32 °C. The adhesive strength was measured with a plate—plate geometry—and a gap of 0.2 mm. The measurements at 25 °C were performed at *t* = 72 h, and the measurements at 32 °C were performed one week after the measurements at 25 °C. The spreadability was evaluated using a simple and reliable technique previously described [24]. Briefly, 1 g of each emulsion was placed at the center of a glass plate. This plate was covered with another glass plate with the same size, and a weight of 200 g was carefully applied on the upper face of the plate. After 1 min, the weight was removed, and the diameter of the spread area (mm) was measured. The measurements were performed in triplicate (*n* = 3).

#### 3.2.4. Validation and/or Exclusion Method

**Figure 5:**
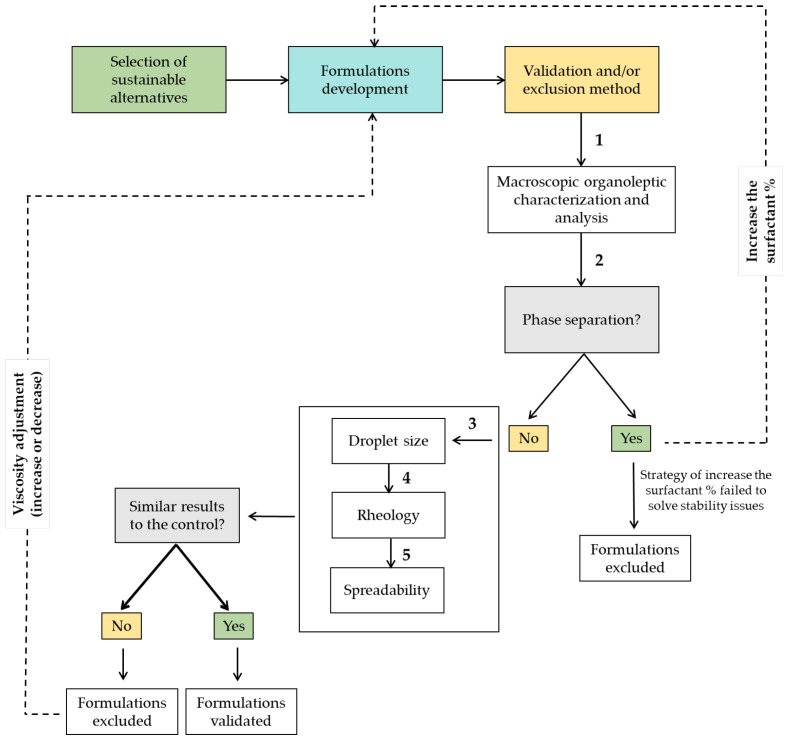
Validation and/or exclusion diagram considering the physicochemical features of O/W emulsions.

A validation and/or exclusion method was performed based on the following sequential tests: (1) macroscopic organoleptic characterization and analysis; (2) physical stability (centrifugation test); (3) droplet size analysis; (4) rheology (rotational and dynamic viscosity measurements, oscillation frequency sweep tests, and evaluation tackiness and adhesion using a pull-away test—adhesive properties); and (5) spreadability. According to the designed decision-making scheme (Figure 5), it is essential to observe that, in the case of phase separation, a strategy of increasing the percentage of surfactant should be used before excluding the formulation. In the cases where the formulations passed the 5 sequential tests but the results were not significantly different from the control, different viscosity adjustment strategies were considered and performed.

#### 3.2.5. Scaling-Up and Stability

##### Manufacturing Process of Scale-Up Formulations

The scale-up production of the emulsions was carried out by increasing the volume of the lab scale by ten-fold (to 1 kg) using a mini-plant reactor system (LR 2 ST, IKA^®^, Staufen, Germany). The emulsions were prepared according to specific manufacturing instructions. Briefly, the aqueous phase was heated at 75 °C and prepared by dispersing the aqueous excipients at 150 rpm inside the reactor vessel. Afterwards, the polymer was dispersed with high shear mixing at a rate of about 8000 rpm/min (T25 ULTRA TURRAX, IKA^®^, Staufen, Germany) for 1 min. The resulting mixture was stirred at 150 rpm until it reached a temperature of 80 °C for 15 min. The oil phase was then prepared and added to the reactor vessel. At this stage, the emulsification phase, a high shear homogenizer, was used for 1 min at 12,000 rpm/min, and, afterwards, the emulsion was homogenized at 250 rpm for 5 min. When the temperature of the emulsion was ~40 °C, the perfume was added, and the stirring was decreased to 150 rpm for 5 min. 

##### Stability

The pilot-scale emulsions were stored during 3 months at 25 ± 2 °C and at 40 ± 2 °C/75% ± 5% relative humidity (RH). Simultaneously, stability was evaluated by simulating the use of the product at 25 °C with application twice a day. Samples were analyzed for pH, macroscopic organoleptic characteristics, physical stability, droplet size, structure, and microbiological control before the storage period and on the first and third storage months, as described in Section 3.2.2. The emulsions were packed in low-density polyethylene (LDPE) tubes (30 g).

#### 3.2.6. Microbiological Control

The efficacy of antimicrobial preservation was assessed using the method recommended by the International Standard ISO 11930:2012(E). Emulsions were contaminated with *Pseudomonas aeruginosa* ATCC^®^ 9027™ (Manassas, VA, USA), *Staphylococcus aureus* ATCC^®^ 6538™ (Manassas, VA, USA), *Candida albicans* ATCC^®^ 10231™ (Manassas, VA, USA), *Aspergillus brasiliensis* ATCC^®^ 16404™ (Manassas, VA, USA), and *Escherichia coli* ATCC^®^ 8739™ (Manassas, VA, USA). Antimicrobial activity was measured throughout the log reduction of the colony-forming units (CFU) by enumeration at time zero and, then, by the monitoring of the kill/survival rate at defined times: 48 h, 7 days, 14 days, and 28 days. A reference sample, an emulsion prepared without preservatives, was used as the negative control.

To select the packaging material, a preliminary study was carried out simulating the use of the product. The aim of this test was to assess the impact of different packaging materials, namely polylactic acid (PLA), polyethylene terephthalate (PET), and glass, on microbiological growth. Briefly, the formulations selected were stored in packages containing the packaging materials under testing, and, once a day, the product was sampled. The microbiological control was carried out taking into account the ISO 16212:2008, ISO 21149:2006, ISO 21148:2005, ISO 17516:2014, ISO 22717:2015, ISO 22718:2006, ISO 18416:2007, and ISO 21150:2015 at 48 h, 7 days, 14 days, and 28 days.

#### 3.2.7. In Vivo Studies 

The protocols for in vivo studies, including the safety, efficacy, and sensorial evaluations, were submitted and approved by the Ethical Committee of PhD Trials^®^ (http://phdtrials.com/, Ethic Committee code, EC 06.01 (ms/2017/4457/P22315), 17th of March), an international contract research organization engaged in the clinical assessment of the safety and efficacy of products for topical application. Besides this approval, it was also ensured that the protocols followed the regulations of the Helsinki Declaration (compliance with good clinical practices) and the Agence Française de Securité Sanitaire des Produits de Santé (AFSSAPS) in order to guarantee that all technical questions were meticulously evaluated during the application of the products in humans.

##### Human Repeat Insult Patch Test (HRIPT)

A safety evaluation study was performed using a Marzulli and Maibach Human Repeat Insult Patch Test (HRIPT) protocol [28]. A panel of 52 healthy volunteers, aged between 21 and 55, were included in the study in which a dermatologist evaluated the irritation/allergic reactions to the tested patches, which were scored according to the International Contact Dermatitis Research Group (ICDRG) [29].

##### Biological Effects

Nine healthy volunteers, aged between 21 and 30, were selected and provided informed written consent. The protocol was approved by the local ethical committee (Lisbon, Portugal). The volunteers applied the two final formulations and the control on the ventral side of the forearm for 28 consecutive days, and the results were compared with a defined control area (anatomically equivalent and without receiving product). The epidermal capacitance was evaluated using two complementary 2D skin capacitance devices—MoistureMap MM 100 and Corneometer^®^ CM 825, respectively—on days 0 and 28. Measurements were performed under standardized conditions at room temperature, according to the Good Clinical Practices rules.

##### Sensorial Analysis

A sensory analysis was conducted according to ISO 11136:2014 using a panel of 9 volunteers. To evaluate the acceptability of the final formulations, a questionnaire was answered by each volunteer to assess sensory attributes such as texture, skin feel on application, hydration, tackiness, odor, spreadability, greasiness, and probability of acquiring the product, using a hedonic scale from 1 to 4 (1—bad, 2—regular, 3—good, and 4—very good). Volunteers were asked to first analyze each product in terms of appearance and smell and, then, to apply the product on the back of the hand in circular movements until disappearance. They were then asked to accurately answer the questions, indicating the option that best-suited their opinion on the proposed scale. The control formulation was also evaluated for comparison purposes. 

## 4. Conclusions

Synthetic ingredients are designed to eliminate certain limitations associated with natural ingredients, usually related to the utilization of innumerous chemical processes, which include synthesis, extraction, and purification methods. Additionally, the use of natural ingredients (i.e., that are directly collected from nature and used in their original state, without any chemical alteration) in the formulation can raise various problems, such as instability and aesthetic limitations. However, the increasing tendency to use green, eco-friendly, and sustainable products justifies the investment on replacement research. Replacement research is a time-consuming process; thus, understanding the chemical and physicochemical properties of raw materials is essential for the formulation process, as it allows predicting stability, performance, and aesthetic challenges. The results obtained in this work show that the replacement of synthetic ingredients by sustainable ones is indeed possible and viable. Several alternatives were studied, and the use of AP, a non-direct substitute for petrolatum, yielded very positive results, supporting that, with adequate strategies of “manipulation” and improvement at the structural and rheological levels, it is possible to overcome certain barriers imposed by the use of natural raw materials. The overall results show that formulations F1 and F2 are suitable for introduction on the market, demonstrating excellent characteristics in terms of physical, chemical, microbiological, rheological, and stability features. The results also show that it is possible to develop appealing sustainable formulations with high standards of quality and marketability.

## Figures and Tables

**Table 1:** Description of the selected petrolatum, dimethicone, and phenoxyethanol alternatives.

Petrolatum Alternatives				Dimethicone Alternatives				Phenoxyethanol Alternatives		
INCI Name	Commercial Name and Abbreviature	Ingredient Category		INCI Name	Commercial Name and Abbreviature	Ingredient Category		INCI Name	Commercial Name and Abbreviature	Ingredient Category
*Ricinus communis* seed oil, Hydrogenated castor oil, *Copernicia cerifera* cera	Natural Vaseline^®^ Type A (NVA)	Petrolatum-like ingredient		Octyldodecyl myristate	MOD (MD)	High viscosity		Benzyl alcohol, Salicylic acid, Glycerin, Sorbic acid	Geogard^®^ ECT (GE)	Cosmetic preservatives
*Butyrospermum parkii*	Massocare^®^ Shea Butter (SB)	Semi-solid butters								
Hydrogenated olive oil, *Olea europaea* fruit oil	Premium Organic Olive Butter^®^ (OB)			Hydrogenated polysobutene	Vitabiosol S (SQ)			Sodium benzoate, Potassium sorbate	Sensicare^®^ C 2010 (SC2)	
*Prunus amygdalus dulcis* oil, Hydrogenated vegetable oil, *Citrus limon* peel oil	Lemon Butter^®^ (LB)									
*Magnifera indica* seed butter	Mango Butter Ultra^®^ (MB)			Hydrogenated ethylhexyl olivate, Hydrogenated olive oil unsaponifiables	Natura-Tec^®^ Plantsil (PLS)	Medium viscosity		Dehydroacetic acid, Benzyl alcohol	Sensicare^®^ C 3000 (SC3)	
*Ricinius communis* seed oil, Hydrogenated *Rhus verniciflua* peel wax, *Rhus succedanea* fruit wax, Ascorbyl palmitate, Tocopherol	Kahl^®^ Vego Jelly 7036 PLUS (KV)	Jelly-like/Blend ingredients								
*Ascorbyl palmitate*, Cera alba, *Copernicia cerifera* cera, *Ricinus communis* seed oil, Tocopherol	Organic Jelly 7236 (OJ)			Propylene glycol dipelargonate	DPPG CG (DPPG)	Low viscosity		Gluconolactone, Sodium benzoate	Geogard Ultra™ (GU)	
PEG-8 Beeswax	Apifil^®^ (AP)	Wax-like ingredient								
Glyceryl dibehenate, Tribehenin, Glyceryl behenate	Compritol^®^ CG 888 Pellets (CM)	Blend ingredients		C15-19 Alkane	Emogreen™ L15 (EMG)			Sorbic acid	(SA)	Food preservatives
C10-18 Triglycerides	Lipocire™ A SG (LPC)									
Jojoba esters, *Helianthus annuus* seed wax, *Acacia decurrens* flower wax, Polyglycerin-3	Acticire^®^ MB (AC)							Sodium benzoate	(SOB)	

INCI: International Nomenclature of Cosmetic Ingredient.

**Table 2:** Adhesive properties at 25 °C and 32 °C (mean ± SD, *n* = 6) and spreadability properties of the formulations (mean ± SD, *n* = 3).

Formulation	Peak Normal Force (N)	Time for Force to Reduce by 90% of Peak(s)	Area under Force Time Curve (N.s)	Diameter of Spread Area (mm)
25 °C				
Control	−0.70 ± 0.13	−0.070 ± 0.013	1.83 ± 0.17	41.0 ± 0.0
F1	−0.78 ± 0.15	−0.078 ± 0.015	1.38 ± 0.35	45.3 ± 0.6
F2	−1.30 ± 0.35	−0.130 ± 0.035	2.62 ± 0.60	40.7 ± 0.6
32 °C				
Control	−0.57 ± 0.10	−0.057 ± 0.010	3.10 ± 0.31	-
F1	−0.60 ± 0.05	−0.060 ± 0.005	4.31 ± 0.12	-
F2	−0.63 ± 0.02	−0.063 ± 0.002	4.82 ± 0.25	-

**Table 3:** Stability test results for formulation F1s and F2s during 3 months at (25 ± 2) °C, at (40 ± 2) °C/(75 ± 5)% relative humidity (RH), and 25 °C (in-use).

Formulation	Conditions of Storage	25 °C		25 °C (In-Use)		40 °C	
	Time (Months)	pH Value	Viscosity (Pa.s) *	pH Value	Viscosity (Pa.s) *	pH Value	Viscosity (Pa.s) *
F1s	0	5.64	15.56	5.65	15.56	5.65	-
	1	5.64	11.32	5.72	12.64	5.72	11.64
	3	5.64	13.88	5.65	18.65	5.80	11.41
F2s	0	5.70	32.32	5.70	32.32	5.70	-
	1	5.70	18.34	5.66	21.29	5.75	16.86
	3	5.70	20.62	5.71	20.46	5.86	22.18

* Apparent viscosity at 2.155 s^−1^.
